# Reintegration needs of young women following genitourinary fistula surgery in Uganda

**DOI:** 10.1007/s00192-019-03896-y

**Published:** 2019-02-27

**Authors:** Alice Emasu, Bonnie Ruder, L. Lewis Wall, Alphonsus Matovu, Godfrey Alia, Justus Kafunjo Barageine

**Affiliations:** 1 TERREWODE Administration, Central Avenue, Soroti, Uganda; 2 Department of Anthropology, Oregon State University, Corvallis, OR USA; 3 Department of Obstetrics & Gynecology, Washington University in St. Louis, St. Louis, MO USA; 4 Department of Anthropology, Campus Box 1114, Washington University in St. Louis, One Brookings Drive, St. Louis, MO 63130-1114 USA; 5 Department of Surgery, Mubende Hospital, Mubende, Uganda; 6 Department of Obstetrics and Gynaecology, Mulago Hospital, Kampala, Uganda; 7 Department of Obstetrics and Gynaecology, Makerere University, Kampala, Uganda; 8 Department of Maternal and Child Health (Save The Mothers), Uganda Christian University, Kampala, Uganda

**Keywords:** Genitourinary fistula, Obstetric fistula, Social reintegration, Uganda, Vesicovaginal fistula, Young women

## Abstract

**Introduction and hypothesis:**

Genitourinary fistulas (usually arising following prolonged obstructed labor) are particularly devastating for women in low-income counties. Surgical repair is often difficult and delayed. While much attention has been devoted to technical surgical issues, the challenges of returning to normal personal, family, and community life after surgical treatment have received less scrutiny from researchers. We surveyed young Ugandan women recovering from genitourinary fistula surgery to assess their social reintegration needs following surgery.

**Methods:**

A cross-sectional survey of 61 young women aged 14–24 years was carried out 6 months postoperatively. Interviews were carried out in local languages using a standardized, interviewer-administered, semistructured questionnaire. Data were entered using EpiData and analyzed using SPSS.

**Results:**

Ongoing reintegration needs fell into interrelated medical, economic, and psychosocial domains. Although >90% of fistulas were closed successfully, more than half of women had medical comorbidities requiring ongoing treatment. Physical limitations, such as foot drop and pelvic muscle dysfunction impacted their ability to work and resume their marital relationships. Anxieties about living arrangements, income, physical strength, future fertility, spouse/partner fidelity and support, and possible economic exploitation were common. Sexual dysfunction after surgery—including dyspareunia, loss of libido, fear of intercourse, and anxieties about the outcome of future pregnancies—negatively impacted women’s relationships and self-esteem.

**Conclusions:**

Young women recovering from genitourinary fistula surgery require individualized assessment of their social reintegration needs. Postoperative social reintegration services must be strengthened to do this effectively.

## Introduction

A female genitourinary fistula is an abnormal communication between the urinary tract and the vagina that results in continuous incontinence. These injuries are rare in affluent countries with well-developed medical systems [1]. When genitourinary fistulas occur in high-income countries, they usually occur as a complication of surgery or cancer and its treatment [1, 2]. A recent Norwegian study of obstetric fistulas found only 40 fistulas related to obstetrics in a cohort of 116,389 deliveries, and of these, only four were genitourinary fistulas and none were from obstructed labor [2]. In contrast, most genitourinary fistulas in low-income lcountries like Uganda are caused by prolonged obstructed labor that results in compression of the vagina, bladder, and other soft tissues by the fetal head against the pubic bone, which leads to tissue necrosis, sloughing, and fistula formation [3, 4]. Fistulas of this type can be prevented by early detection of obstructed labor and prompt intervention (cesarean delivery) to prevent the injury from progressing to the point of tissue necrosis; however, this is not possible without a healthcare delivery system that functions efficiently and effectively [3, 4]. When cesarean section is performed under difficult conditions, the operation itself may result in a genitourinary fistula [1, 3, 4].

The incidence and prevalence of genitourinary fistulas in Uganda are imprecisely known due to reporting deficiencies in the health system, but such injuries appear to be relatively common [5, 6]. This malady has serious consequences for women who are affected [3–5, 7, 8]. The injury impacts their physical and psychosocial health, marriages, sexuality, family life, economic well-being, and ability to function as productive members of the community. Obtaining effective surgical treatment for these injuries is often difficult and delayed [9, 10], and readjustment to social life afterwards is problematic [13–16].

We carried out a cross-sectional survey of young women recovering from genitourinary fistula surgery in Uganda to assess their rehabilitation and social reintegration needs in order to strengthen the ongoing reintegration policy and program for women recovering from fistula.

## Materials and methods

A cross-sectional sample of 61 young women (aged 14–24 years) who had undergone surgery for genitourinary fistula was recruited 6 months postoperatively to determine their reintegration needs. Recruitment was carried out by TERREWODE, a grassroots Ugandan women’s empowerment organization that focuses on obstetric fistula as a primary social and human rights concern. Individuals were interviewed in their home communities after having undergone fistula repair surgery at various clinical sites within Uganda (Mulago, Mubende, Mbale, Soroti, Arua, Lira, and Kitovu hospitals). All women provided written informed consent (signature, or thumbprint if illiterate) to participate in the study, and their ages were verified by recall and/or through their Ugandan national identity cards. All participants were interviewed in their local languages (Luganda, Ateso, Luo, and Lugbara) by native speakers using a standardized semistructured questionnaire. Interviews took place from 1 April to 31 August 2017. Participants were compensated the equivalent of US $12 to cover the costs of transportation and refreshments.

Data on sociodemographic characteristics, obstetric history, health concerns, psychosocial needs, sexual and reproductive worries, and economic issues were collected, coded, entered into a computerized database using EpiData version 3.1 (EpiData Association, Odense, Denmark, 2000–2018) and then exported to SPSS software (Version 14, Chicago, IL, USA, SPSS Inc., 2005) for analysis. Completed questionnaires were stored securely to maintain confidentiality. Ethical clearance was obtained from the Makerere University School of Medicine Research Ethics Committee in Kampala, Uganda (Reference No. 2017–54). The questionnaire was pretested in the respective local communities based on study languages used. During the data collection process, the first author double-checked each questionnaire for completeness and accuracy at the time of data collection. Daily meetings were held by supervisors during data collection to ascertain its validity.

## Results

Demographic characteristics of the study population are provided in Table 1. The average age was 21.6 (range 14–24) years. Two thirds had some primary school education, half were literate in their local language, and half were literate in English. Most were Christian of various denominations; a small number were Muslims. One third were currently married, suggesting the enormous social impact that developing a fistula may have on intimate family institutions.

**Table 1:** Sociodemographic characteristics of participants

Variable		Number (%)
Age	Mean age: 21.6 (± 2.6), range: 14–24 years	61 (100)
Age category		
• >18 years		6 (9.8)
• 18–24 years		55 (91.2)
Education		
• None		2 (3.3)
• Partial or complete primary school		49 (80.3
• Secondary (O level)		10 (16.4)
Literate in local language		
• Yes		31 (50.8)
• No		30 (49.2)
Literate in English		
• Yes		24 (39.3)
• No		37 (60.7)
Religion		
• Roman Catholic		23 (37.7)
• Protestant Christian		22 (36.1)
• Charismatic/Pentecostal Christian		8 (13.1)
• Muslim		8 (13.1)
Current marital status		
• Never married		10 (16.4)
• Currently married		20 (32.8)
• Cohabiting with a partner		30 (49.2)
• Data missing		1 (1.6)
Type of marriage (among those currently married, *n* = 20)		
• Customary marriage with bridewealth payments^a^		17 (85.0)
• Islamic marriage		3 (15.0)
Polygamous marriage (among those currently married, *n* = 20)		
• Yes		6 (30.0)
• No		13 (65.0)
• Data missing		1 (5.0)
Number of wives (among polygamous marriages, *n* = 6)		
• Two		4 (66.7)
• Three		1 (16.7)
• Four		1 (16.7)
Change in marital status due to fistula (among those currently married or cohabiting)		
• Yes		31 (62.0)
• No		19 (38.0)

^a^Marriage payments are commonly referred to as dowry but is actually a payment made by the family of the groom to the family of the bride. In anthropology this is more properly called bridewealth, and we kept this more technical term for this reason

Obstetric characteristics related to fistula development are given in Table 2. Obstetric causes accounted for 53 cases, six cases were due to other forms of trauma (including road traffic accidents and surgical misadventure), and in two cases, the cause of the injury was obscure. Most women developed their fistulas early (mean 18.8 years), and slightly more than half developed the fistula during their first pregnancy (28 cases, 52%). The average reported duration of labor for obstetric fistulas was 2.6 days (range 1–7 days), and 70% of women had either a stillbirth (26 cases) or a neonatal death (11 cases). Only 16 women (30%) had a living child following their delivery. Most women (36 cases, 68%) underwent delivery by cesarean section. It cannot be determined how many (if any) fistulas developed as a direct result of an injury sustained at the time of surgery. Because the average reported duration of labor was nearly 3 days, it is likely that pressure necrosis had already occurred at the time of cesarean section, but in some cases, damaged tissues might have been injured further at operation, as many of these cases would have been surgically challenging.

**Table 2:** Fistula history

Variable	Number (%)
Age at which fistula occurred: 18.8 (± 3.8), range 14–24 years	61 (100)
Duration of fistula at the time of repair	
• < 1 year	26 (42.6)
• 1–5 years	23 (37.7)
• > 5 years	9 (14.8)
• Missing data	3 (4.9)
Perceived cause of fistula	
• Obstetric trauma	53 (86.9)
• Other forms of trauma, including accidents and surgical injury	6 (9.8)
• Unspecified	2 (3.3)
Pregnancy in which obstetric fistula occurred (*n* = 53)	
• First	28 (52.8)
• Second	14 (26.4)
• Third	4 (7.6)
• Fourth or higher	7 (13.2)
Reported duration of labor (for cases of obstetric fistula) 2.6 (± 1.5) days	
Outcome of delivery leading to fistula (*n* = 53)	
• Stillbirth	26 (49.1)
• Neonatal death	11 (20.8)
• Living child	16 (30.2)
Type of delivery at pregnancy leading to fistula (*n* = 53)	
• Spontaneous vaginal delivery	8 (15.1)
• Operative vaginal delivery	9 (20.8)
• Cesarean delivery	36 (67.9)
Location of delivery leading to fistula (*n* = 53)	
• Home delivery	4 (7.6)
• Private clinic	3 (5.7)
• Health center (level 2 or 3)^a^	9 (17.0)
• Health center (level 4, district hospital)	16 (30.2)
• Regional referral hospital	20 (37.7)
• Private hospital	1 (1.9)

^a^Health centre 2 is a parish-level, basic primary health facility. Health center 3 is a subcounty basic primary health facility providing slightly more advanced care. Both are equipped for basic deliveries but neither provide comprehensive emergency obstetric care

Delays in receiving care were common. Less than half of women with a fistula (26 cases, 43%) were able to get treatment within the first year after injury. Only 21 women (40%) had no additional health problems; the rest experienced a variety of comorbidities after delivery, including foot drop from nerve injury during prolonged labor (13 cases, 24.5%), menstrual irregularity (4 cases), dyspareunia (3 cases), urinary tract infection (3 cases), hysterectomy (3 cases), and sepsis (1 case). Ongoing health concerns were also common, including persistent pain (back, chest, abdomen, pelvis; 13 cases, 21%), genital ulcers (8 cases; 13%), urinary tract infections (4 cases; 7%), kidney disease and HIV/AIDS (2 cases each; 3% each), and depression, asthma, hypertension, toothache, other sexually transmitted diseases, amenorrhea, and psychological abuse (1 case each).

Health-seeking behavior was common, and a variety of sources of care were utilized. Biomedical help was sought by virtually all patients (Table 3), but many women used traditional herbal healers (11 cases; 18%), spiritual healers (5 cases; 8%), churches (2 cases; 3%), and family members (3 cases; 5%). Only four fistula cases (7%) were treated unsuccessfully. Initial surgery was successful in 51 cases (84%), and six additional fistulas (10%) were successfully closed after multiple surgeries. Almost half the patients (28 cases; 46%) reported ongoing health concerns after fistula repair. Of those with persistent needs, 19 (68%) required ongoing financial support, eight (25%) had counseling and educational needs, and ten (36%) required ongoing medical treatment for various conditions. Seventeen women (28%) had had another pregnancy since developing their fistula, resulting in four miscarriages (24%), 12 live births, and one ongoing pregnancy at the time of interview.

**Table 3:** Health concerns following fistula development

Variable	Number (%)
Type of help sought because of fistula (more than 1 answer possible)	
• Traditional healers	11 (18.0)
• Prayer from spiritual healers (including churches)	7 (11.5)
• Medical therapy (before surgical intervention)	60 (98.4)
• Family remedies	3 (4.9)
• Herbal medicine	2 (3.3)
Ultimate outcome of fistula treatment	
• Unsuccessful	4 (6.6)
• Successful at initial treatment	51 (83.6)
• Successful after multiple surgeries	6 (9.8)
Number of fistula surgeries	
• 1	53 (86.9)
• 2	4 (6.6)
• 3	3 (4.9)
• ≥4	1 (1.6)
Pregnancy since fistula	
• Yes	17 (27.8)
• No	44 (71.2)
Outcome of pregnancy since fistula (*n* = 17)	
• Miscarriage	4 (23.5)
• Live birth	12 (70.6)
• Pregnant at the time of interview	1 (5.9)
Ongoing health concerns after fistula	
• Yes	28 (45.9)
• No	33 (54.1)
Perceived needs to help manage ongoing health conditions/concerns (*n* = 28)	
• Financial support	19 (67.9)
• Education or counseling	7 (25.0)
• Family counseling	1 (3.6)
• Medical treatment	10 (35.7)

The presence of a fistula created significant psychosocial burdens for these women (Table 4). Nearly all expressed uncertainty and fearfulness at the time of hospital discharge (56 cases; 95%). Most women returned to live with their parents afterward (35 cases; 57%) or returned to be with a partner (15 cases; 25%). The rest dispersed to guardians, other family members, or were uncertain or confused about their plans (2 each). Nearly all women acknowledged the presence of factors that would hinder or prevent their return to normal life after surgery (59 cases; 97%).

**Table 4:** Psychosocial needs related to fistula care

Variable	Number (%)
Fearful at time of hospital discharge	
• Yes	58 (95.0)
• No	3 (5.0)
Plans for residence after hospital discharge	
• Parents	35 (57.4)
• Partner	15 (24.6)
• Guardian	2 (3.3)
• Other family members	2 (3.3)
• Confused or uncertain about plans	2 (3.3)
• Missing data	2 (3.3)
Presence of factors limiting return to normal life	
• Yes	59 (96.7)
• No	2 (3.3)
Partner’s reaction (for those with a partner; *n* = 53) (multiple responses possible)	
• Abandoned, separated, or dismissed by partner	25 (47.2)
• Partner emotionally or physically abusive	12 (22.6)
• Partner brought in another wife	9 (17.0)
• Partner unsupportive, but did not separate or abandon	9 (17.0)
• Partner remained engaged and supportive	8 (15.1)
• Partner unfaithful after fistula	6 (11.3)
Plans for further education after fistula	
• Yes	51 (83.6)
• No	10 (16.4)
Those who visited the patient while under treatment (more than 1 answer possible)	
• Friends, neighbors, and community members	21 (34.4)
• Parents	13 (21.3)
• Church members and religious leaders	7 (11.5)
• Siblings and other relatives	40 (65.6)
• In-laws	9 (14.8)
• Members of TERREWODE	7 (11.5)
• Husband/partner/boyfriend	5 (8.2)
• Nurse	1 (1.6)
• No one	12 (19.7)

Social stresses relating to partners were particularly prevalent following a fistula. Of the 53 women who were in an ongoing relationship, 25 (47%) reported having been abandoned, separated, or driven away by their partner. In 12 cases (23%), their partners were emotionally or physically abusive. In nine cases (17%), their partner took another wife after the fistula occurred, and in six cases (11%), the fistula resulted in sexual infidelity on the part of their partners. Only eight women (15%) reported that their partner provided them with ongoing support. A further nine women (17%) reported that although they regarded their partners as generally unsupportive, they continued to be in a relationship and had not been totally abandoned. One partner developed clinical depression in the fistula’s aftermath, increasing the psychosocial stress in the relationship.

While under treatment, women with genitourinary fistulas reported varying levels of social support from visitors. Only 12 women (20%) reported being completely alone and isolated, with no visitors. Somewhat surprisingly, only five women (8%) reported that they were visited by their husband, partner, or boyfriend during treatment. The most common visitors were family members: parents (13; 21%), siblings and other relatives (40; 66%). Friends, neighbors, and members of the local community (21; 34%) were the most common visitors, but visits were also made by church members and religious leaders (7; 11%), TERREWODE staff (7; 12%), and nurses (1; 2%). The vast majority of women (56, 92%) received assistance from someone; only five (8%) reported receiving no help of any kind. These data suggest that women with fistulas remain within social networks after sustaining their injuries, but their lives become more difficult, and social stress increases dramatically.

Women reported numerous sexual and reproductive health concerns following fistula surgery (Table 5). The overwhelming majority of women declared their desire for further children (52; 85%), and half (29; 48%) wanted to use family planning methods to space a pregnancy appropriately. However, only 16 (26%) were using family planning at the time of interview, and only two thirds (40; 66%) reported having had family planning discussed with them following their fistula repair.

**Table 5:** Sexual and reproductive needs after fistula

Variable	Number (%)
Discussed family planning after fistula surgery	
• Yes	40 (65.6)
• No	21 (34.4)
Wish to have more children	
• Yes	52 (85.3)
• No	9 (14.8)
Currently using family planning method	
• Yes	16 (26.2)
• No	45 (73.8)
Would like to use family planning to space or limit children	
• No	24 (39.3)
• Yes	29 (47.5)
• Not applicable	5 (8.2)
• Missing data	3 (4.9)
Currently in an intimate relationship	
• Yes	38 (62.3)
• No	23 (37.7)
Would like to be in an intimate relationship (*n* = 23)	
• Yes	7 (30.4)
• No	16 (69.6)
Intimate partner the same as before fistula occurrence	
• Yes	27 (71.1)
• No	11 (29.7)
Difficulties with sexual intercourse after fistula	
• No	31 (50.8)
• Painful	8 (14.8)
• Afraid or fearful of intercourse	4 (6.6)
• Loss of sexual interest	9 (14.8)
• Not applicable	9 (14.8)

After hospital discharge, 38 women (62%) were in an intimate relationship. Of the 23 women who were not in a relationship, seven (30%) expressed a desire to have such a relationship in the future. Of those with current intimate partners, 27 (71%) had the same partner as before the fistula; 11 (29%) were in a new relationship after treatment. Half of women reported satisfactory sexual intercourse after fistula surgery (31; 51%). For eight women (15%), intercourse was painful; four (7%) expressed fear concerning intercourse; nine (15%) had lost interest in sex altogether; nine were not sexually active at the time of interview.

Economic insecurity was one of the biggest problems for these young women after being stricken with a fistula (Table 6). Most (41; 69%) were unemployed prior to their injuries. Those who were employed worked in a variety of different occupations, including agriculture or quarrying, hairdressing, house cleaning, petty trading, assisting in a shop, or were self-employed in minor businesses. Development of a fistula was economically devastating: only two women were able to continue working afterward. The rest lost their jobs or their businesses or were simply too debilitated to continue working. Of the 61 women, 14 (23%) were employed for pay after fistula surgery. Of those employed, none earned >50,000 Ugandan shillings per week (US$13.50; US$1 = 3690 Ugandan shillings), meaning that even if employed, these women were all living on <US$2.00 per day. Only one woman said this was enough to meet her basic needs, and one reported being able to save anything at all from her income.

**Table 6:** Socioeconomic status and needs related to fistula

Variable	Number (%)
Employed before fistula	
• Yes	19 (31.2)
• No	41 (68.9)
Type of job before fistula	
• Agriculture or quarrying	3 (15.8)
• Hair salon	1 (5.3)
• Housekeeper or house cleaner	2 (10.5)
• Self-employed	2 (10.5)
• Petty trading—selling groundnuts, charcoal, etc.	4 (21.1)
• Shop assistant	4 (21.1)
• Missing/incomplete	3 (15.8)
How fistula affected employment	
• Lost business	8 (57.9)
• Lost job	4 (21.1)
• Continued working	2 (10.5)
• Stopped working/too weak	6 (31.6)
Currently employed for pay	
• Yes	14 (23.0)
• No	43 (70.5)
• Missing data	4 (6.6)
Current weekly income if employed (*n* = 14) (US$1 = 3690 Ugandan shillings)	
• < 5000 Ugandan shillings	2 (14.3)
• 5000–10,000 Ugandan shillings	5 (25.7)
• 11,000–20,000 Ugandan shillings	2 (14.3)
• 21,000–30,000 Ugandan shillings	1 (7.1)
• 31,000–40,000 Ugandan shillings	1 (7.1)
• 41,000–50,000 Ugandan shillings	1 (7.1)
• Missing data	2 (14.3)
Borrowed money or used savings to cover household costs in past month	
• Yes	31 (50.8)
• No	29 (47.5)
• Missing data/incomplete	1 (1.6)
Experienced economic abuse before fistula	
• Yes	30 (49.2)
• No	31 (50.8)
Experienced economic abuse after fistula	
• Yes	39 (65.0)
• No	20 (33.3)
• Missing data	1 (1.7)

Economic abuse by others was a common experience for these women, even before the fistula (30; 49%), but the prevalence of such economic exploitation continued and even increased significantly after their injuries (39; 65%). Abuses included the sale of agricultural crops for which they were not paid, refusal to pay for services or products they had provided to others, theft/sale of animals, denial of land use, and nonrepayment of debts owed. A quarter of these women suffered economic abuse after a fistula at the hands of their husbands (10; 26%); others were exploited by their parents (6; 15%), community groups (5; 13%), other relatives including siblings and in-laws (6; 15%), or their employers (4; 10%). Many women lost access or user rights to property (18; 30%), lost decision-making power (11; 18%), and many reported stigmatization, loss of respect, erosion of trust, and lack of consideration by others (7; 18%).

## Discussion

The survey provided a robust picture of young Ugandan women recovering from genitourinary fistula surgery, including the impact of the malady and the socioeconomic needs it created. It documents the persistent needs faced by young Ugandan women after treatment of genitourinary fistulas. These ongoing needs fall into broad medical, economic, and psychosexual categories. The overwhelming majority of fistulas were the result of obstetrical trauma from prolonged obstructed labor. Although the fistulas themselves were repaired successfully in >90% of cases, almost half of women reported ongoing health concerns afterward, findings similar to those from other studies [15–17]. A third of women required ongoing medical treatment for comorbidities, and another third felt the need for continued personal or family counseling. A quarter of these women (13) developed foot drop from nerve compression injuries during labor, which could potentially affect their physical ability to earn a living in the future [16, 19].

Because motherhood is regarded as an essential component of the social identity and self-esteem of Ugandan women, it is not surprising that 85% of these young women expressed the desire for future childbearing. Fears pertaining to future reproductive success and worries about developing a recurrent fistula are common in women who have had an obstetric fistula [11–13, 15, 16, 20, 21]. There is considerable anxiety surrounding whether their future fertility would be impaired after fistula surgery. Most reports show fairly dismal subsequent fertility after sustaining a fistula [20–22]. In this survey, 26% of women were using any form of family planning although most were in an intimate relationship, yet only 17 (28%) had become pregnant following surgery, resulting in 12 live births. Future reproductive success will likely be a persistent concern for these women for many years.

The economic needs of these women after fistula repair were notable. This is a persistent theme among women recovering from fistula surgery [12, 13]. As a group, they were economically vulnerable even before they developed a fistula. Most were unemployed other than as homemakers; 31% (19) had other employment. For employed women, the fistula was a devastating blow: 80% (12) lost their jobs or their businesses as a result of their condition, and only 14 (23%) were employed after fistula surgery. Virtually all women were struggling economically, even after fistula repair. Half had to borrow money or use up their savings to meet their living expenses. Although half reported suffering some form of economic abuse before their injury, nearly two thirds reported being victims of economic exploitation afterward.

The medical and economic needs of these women were closely tied to their psychosocial needs. The development of a vesicovaginal fistula is a striking blow to a woman’s view of her own self-worth. Numerous studies have documented high rates of shame, depression, and feelings of stigma [5, 7, 8, 11, 12, 14–18]. Psychological blows, combined with lost economic productivity, overwhelming personal hygiene needs, frequent absences occasioned by the need to seek medical care, and impaired sexuality from childbirth injuries, introduce powerful stressors into marital relationships, which often crumble under their impact, particularly if there are no living children from the union [4, 5, 7–11, 14–18, 23]. This leads to high rates of separation and divorce, which are also seen in this study. Even when the fistula is repaired successfully, prior personal and marital relationships may not heal. Some women are reluctant even to try to re-establish intimate relationships. In this study, almost 40% (23) were not in such a relationship after repair, and 16 had abandoned the desire to find a partner; 27 (44%) had the same intimate partner after surgery as before developing a fistula.

Sexual dysfunction was particularly common following vesicovaginal fistula repair. This finding has also been reported in prior studies [23–25]. Half the women in our study reported no difficulties with sexual intercourse; 15% lost interest in sex, 15% experienced regular pain with intercourse, ~7% were afraid of intercourse, and 38% were not in an intimate relationship. Multiple factors were probably involved in this sexual dysfunction, including vaginal scarring from obstructed labor and surgery, pelvic-muscle-disuse atrophy and incoordination, and an unconscious guarding reflex following painful intercourse (vaginismus). These physical injuries (including foot drop) highlight the importance of pelvic muscle rehabilitation as a crucial component of ongoing fistula care.

Based on this survey of the needs of young women following repair of genitourinary fistulas in Uganda, we recommend that institutions providing surgical services for this condition conduct preoperative screening of patients’ medical condition, economic status, and psychosocial circumstances in order to develop individualized plans for postoperative rehabilitation and reintegration. The services most likely to be needed by such women are help in finding a place to live after surgery, development of a social support network (often composed of other fistula survivors), access to health services to meet ongoing medical needs, psychological and family counseling, and economic support and skills development. There is good evidence that successful fistula repair is associated with marked improvements in mental health, but many patients will benefit from individual psychological counseling and group psychotherapy during the recovery period [14, 24–28]. Economic support might include cash grants, inclusion in microfinancing schemes, job skills training (such as small business development, craft training, formation of economic cooperatives, etc). Training in advocacy for women’s health and justice issues will provide psychosocial support as well as increase the platform of public awareness concerning the medical and social issues around obstetric fistula formation (i.e., lack of accessible services, lack of transportation, poor-quality obstetric care in health facilities, etc).

The high level of sexual dysfunction reported following surgery can be addressed in part by making sure that fistula centers provide high-quality physical therapy services specifically directed at the pelvic floor. The potential of such services to improve the quality of life of women who have sustained catastrophic childbirth injuries is likely to be substantial [24, 28].

The strengths of this paper include data acquisition carried out by native speakers of indigenous Ugandan languages, grass-roots familiarity with the local communities affected by obstetric fistula, and a relatively large and diverse patient population taken from across Uganda. Weaknesses include possible selection bias in patient recruitment. Since the study sample was limited to young women, our findings may not be generalizable to a larger population of older women with genitourinary fistulas who undergo surgical repair. The study adopted a nonprobability consecutive sample that is not necessarily a truly representative statistical sample. This means that results may not be generalizable to the broader population of young women affected by fistula. The cross-sectional nature of the survey may also mean that results could vary over time.

Reintegration into society after genitourinary fistula surgery in Uganda requires an individualized approach to each woman, paying attention to her ongoing medical, economic, and psychosocial needs. Such programs must be incorporated into the overall care of fistula patients if the best possible outcomes are to be achieved.
